# Modulation of *R*-gene expression across environments

**DOI:** 10.1093/jxb/erv530

**Published:** 2016-03-15

**Authors:** Alice MacQueen, Joy Bergelson

**Affiliations:** Department of Ecology and Evolution, University of Chicago, 1101 East 57th Street, Chicago, IL 60637, USA.

**Keywords:** *Arabidopsis thaliana*, climate, disease resistance, environmental stress, gene expression, natural variation, plasticity, *R*-gene.

## Abstract

Some environments are more conducive to pathogen growth than others, and, as a consequence, plants might be expected to invest more in resistance when pathogen growth is favored. Resistance (*R*-) genes in *Arabidopsis thaliana* have unusually extensive variation in basal expression when comparing the same *R*-gene among accessions collected from different environments. *R*-gene expression variation was characterized to explore whether *R*-gene expression is up-regulated in environments favoring pathogen proliferation and down-regulated when risks of infection are low; down-regulation would follow if costs of *R*-gene expression negatively impact plant fitness in the absence of disease. Quantitative reverse transcription–PCR was used to quantify the expression of 13 *R*-gene loci in plants grown in eight environmental conditions for each of 12 *A. thaliana* accessions, and large effects of the environment on *R*-gene expression were found. Surprisingly, almost every change in the environment—be it a change in biotic or abiotic conditions—led to an increase in *R*-gene expression, a response that was distinct from the average transcriptome response and from that of other stress response genes. These changes in expression are functional in that environmental change prior to infection affected levels of specific disease resistance to isolates of *Pseudomonas syringae*. In addition, there are strong latitudinal clines in basal *R*-gene expression and clines in *R*-gene expression plasticity correlated with drought and high temperatures. These results suggest that variation in *R*-gene expression across environments may be shaped by natural selection to reduce fitness costs of *R*-gene expression in permissive or predictable environments.

## Highlight

*R*-gene expression strongly affects plant fitness and biotic resistance and varies significantly, with short-term and historic environment in patterns distinct from the transcriptome, consistent with selection driving *R*-gene expression variation.

## Introduction

Plants in natural environments are frequently exposed to multiple stresses simultaneously. Plants exposed to combinations of stresses respond in ways that are distinct from, and in some cases entirely unpredictable based on gene expression in response to each stress alone (Suzuki *et al.*, 2014). Expression of defense-related genes may vary with the abiotic environment due to associated changes in the probability and severity of disease. For example, high humidity and low temperatures promote more severe fungal outbreaks (Holub *et al.*, 1994), while outbreaks of viral diseases are associated with cold snaps (Szittya *et al.*, 2003). Plants grown in environments prone to infection may thus invest more in the expression of defense response genes.

Resistance genes (*R*-genes) are a vital component of the plant immune system that function by producing proteins that recognize, directly or indirectly, specific effectors secreted by a pathogen. This recognition event, termed the gene-for-gene interaction, is highly specific and leads to the induction of a robust defense response. Despite the intuition that surveillance genes should be consistently expressed at low levels to allow pathogen detection, *Arabidopsis thaliana* genotypes vary extensively in the basal expression of most *R*-genes. In a survey of expression of 45 gene families measured in 19 accessions of *A. thaliana*, the two *R*-gene subfamilies were in the top three families of differentially expressed genes (Gan *et al.*, 2011). Indeed, the extent of differential expression for *R*-genes can be impressive, with up to 350-fold differences between accessions, the highest for any gene.

Natural variation in gene expression has been previously associated with important phenotypic variation (Baxter *et al.*, 2010; Chan *et al.*, 2011; Li *et al.*, 2011; Studer *et al.*, 2011). However, it is surprising that *R*-gene expression is so variable between populations, given the large, pathogen-dependent trade-offs in fitness associated with differences in *R*-gene expression. Plants with specific *R*-genes are up to 10% less fit than plants lacking that *R*-gene when grown in the absence of targeted pathogens (Tian *et al.*, 2003; Karasov *et al.*, 2014). Fitness costs are further evident in the dwarfing that results when plant mutants constitutively overexpress *R*-genes; of 12 *R*-genes that have been overexpressed or constitutively activated in *A. thaliana*, two are lethal and nine carry significant growth defects (Mindrinos *et al.*, 1994; Shah *et al.*, 1999; Shirano *et al.*, 2002; Stokes *et al.*, 2002; Xiao *et al.*, 2002; Grant *et al.*, 2003; Igari *et al.*, 2008; Aboul-Soud *et al*., 2009; Palma *et al.*, 2010; Kato *et al.*, 2011; Boccara *et al*., 2014; Kato *et al.*, 2014). An additional study increased expression without misexpression by introducing multiple copies of two *R*-genes under control of their native promoters. They found that increased expression decreased plant size (Xiao *et al.* 2002), presumably as a consequence of increased metabolic burden. Even a 2-fold increase in expression of a single *R*-gene caused dwarfism and reduced seed set (Xiao *et al.*, 2002); basal *R*-gene expression varies even more than this between populations of *A. thaliana*.

The benefits of recognizing secreted effectors counteract these costs and potentially select for this expression variation. Fitness benefits of up to 30% have been demonstrated for plants capable of recognizing a particular *Pseudomonas syringae* isolate relative to plants that cannot (Gao *et al.*, 2009). A link between *R*-gene expression variation and fitness benefits of resistance depends on whether increases in basal *R*-gene expression provide more effective defense. This question has been addressed in two ways. First, seven studies have used mutants to ask whether basal *R*-gene overexpression impacts defense against pathogens in *A. thaliana*. Six of these studies found that increased expression led to a significant decrease in pathogen load (Shah *et al.*, 1999; Stokes *et al.*, 2002; Grant *et al.*, 2003; Aboul-Soud *et al*., 2009; Kato *et al.*, 2011; Boccara *et al*., 2014; but see Shirano *et al.*, 2002). In a more natural context, three studies have tested the effect of *R*-gene expression in *A. thaliana* on defense against specific pathogens; two of these studies found that increased expression led to a significant decrease in pathogen load (Van Poecke *et al.*, 2007; Zhang and Gassmann, 2007; Boccara *et al*., 2014). Thus, most studies support a clear link between *R*-gene expression and resistance to pathogens.

The net effect of *R*-gene expression on plant fitness clearly depends on the distribution of pathogens through time and space. Additionally, if the costs and benefits of *R*-gene expression vary as a function of the abiotic environment, selection may favor different levels of *R*-gene expression in different environments. Indeed, a genome-wide association study (GWAS) supports an interaction between *R*-genes, climate, and pathogen load (Hancock *et al.*, 2011). In this study, single nucleotide polymorphisms (SNPs) associated with a suite of five climate variables overlapped with four quantitative trait loci (QTLs) associated with proliferation of the bacterial pathogen *P. syringae* DC3000. These climate variables (minimum temperature in the coldest month, number of consecutive cold days, relative humidity in spring, temperature seasonality, and precipitation in the driest month) may select for higher *R*-gene expression levels as they are likely to increase the probability that *P. syringae* infects its hosts (Underwood *et al.*, 2007; Ferrante *et al.*, 2012). Results from the study of Hancock *et al.* (2011) also identified a SNP within the coding region of an *R*-gene (At5g22690) that was significantly correlated with both precipitation in the driest month and relative humidity in the spring, implying that the distribution of *R*-gene alleles may be shaped by climate.

The pathogens recognized by most *R*-genes are unknown, making the environments favoring their proliferation necessarily unknown. To explore if and how plants modulate their *R*-gene expression in response to risk of infection, effects of short-term environment and historical climate on *R*-gene expression were determined. These environments were selected to represent environmental conditions likely to impact pathogen proliferation. If optimized, *R*-gene expression levels should track relative risks, increasing when the risk of infection is high, in cold and wet environments, and decreasing when it is low, in hot and dry environments. Alternatively, environmental perturbations themselves may provide sufficient reason to enhance levels of defense. Since plants contain diverse microbial communities (Bodenhausen *et al.*, 2013; Turner *et al.*, 2013; Horton *et al.*, 2014), and since plant pathogens carrying *R*-gene-recognized avirulence genes include multiple taxonomic domains, the outcome of pathogen infection may be best understood as a community assembly problem, where the pathogens are invasive species (Costello *et al.*, 2012). Pathogens are then likely to cause disease in conditions that help species invade and proliferate; that is, disturbed conditions to which the community is not well adapted (Shea and Chesson, 2002).

Here, diverse accessions of *A. thaliana* were grown in a set of eight environmental conditions to explore the relationships between abiotic perturbations, historical climate, and *R*-gene expression patterns under controlled experimental conditions. The expression of 13 *R*-genes in each of 12 *A. thaliana* accessions from disparate historical climates was determined. First, the average effect of current environment on *R*-gene expression and the effect of environment on the resistance response in one of these environments were measured. *R*-genes were up-regulated on average in response to all abiotic perturbations, with extensive variation in *R*-gene expression between accessions. A meta-study of the effect of 15 publicly available biotic and abiotic treatments on *R*-gene expression in *A. thaliana* demonstrated that the *R*-gene response to a variety of biotic perturbations differed from the responses to both non-*R*-genes and other stress response genes. Functional resistance increased after an environmental perturbation followed by infection with *P*. *syringae*. Having found substantial variation in *R*-gene expression across environmental treatments, the geographical distribution of *R*-gene expression was examined. In particular, the expression of the 12 *A. thaliana* accessions was examined for the presence of environment-of-origin clines in both *R*-gene expression and plasticity in *R*-gene expression. RNA sequencing (RNA-seq) data from 14 worldwide *A. thaliana* accessions and 144 Swedish *A*. *thaliana* accessions was examined to confirm these clines. In combination, these two sets of analyses allowed the investigation of the effect of environment on expression and the likelihood that variation in *R*-gene expression is shaped by the distribution of pathogens or other aspects of adaptation to the local environment.

## Materials and methods

### Expression study: experimental strategy and overview

The experimental design included 12 *A. thaliana* accessions (Table 1) selected from a broad geographical distribution across Europe and a latitudinal cline in the Midwest USA. For each of these accessions, quantitative PCR (qPCR) was used to measure expression of 13 randomly selected *R*-genes in control conditions, and after seven abiotic perturbations (Supplementary Data set S1 available at *JXB* online). Relative expression levels for these seven perturbations were considered relative to one unmanipulated control. Each perturbation was motivated by the temperature and precipitation climate variables associated with pathogen growth and recognition identified in Hancock *et al.* (2011). Since transcriptional responses to perturbations of different lengths can differ, two treatment durations were applied. Perturbations included 3h of heat shock and cold shock, as well as week-long changes to short days, high temperatures, low water, high temperature and low water, and high humidity. Due to space constraints, a single perturbation, the week-long change in day length, was manipulated for the Midwestern cline (Table 1) as this was the variable most likely to correlate with latitude. *R*-genes were chosen to represent the entire set and included singletons, genes from clusters, and genes in each of the major *R*-gene subfamilies (Supplementary Table S1). Comparisons among the 12 accessions allowed the exploration of how historical climate impacted patterns of basal *R*-gene expression, how *R*-gene expression was modulated by weather (abiotic perturbations), and how historical climate shaped these *R*-gene responses.

**Table 1.** Accessions used for the expression study with details on their location of origin, treatments used, and the reasoning behind the accession’s inclusion

Name	Ecotype ID	Location of origin	Treatments	Reasoning
Col-0	8279	Midwest USA	1–8	Clark *et al.* (2007) diverse set
Cvi-0	8281	Canary Islands	1–8	Clark *et al.* (2007) diverse set
Est-1	8291	Estonia	1–8	Clark *et al.* (2007) diverse set
Fei-0	8294	Portugal	1–8	Clark *et al.* (2007) diverse set
Kin-0	8316	MI, USA	1–2	Latitudinal cline
Kno-11	6810	IN, USA	1–2	Latitudinal cline
Ler-1	8324	Germany	1–8	Clark *et al.* (2007) diverse set
Rmx-A02	8370	MI, USA	1–2	Latitudinal cline
RRS-7	8373	MI, USA	1–8	Clark *et al.* (2007) diverse set and in latitudinal cline
SLSP-30	2274	MI, USA	1–2	Latitudinal cline
Tsu-1	8394	Italy	1–2	Clark *et al.* (2007) diverse set
Van-0	8400	Western Canada	1–8	Clark *et al.* (2007) diverse set

### Plant materials and growth conditions

Seeds from each accession were sown in 50:50 Farfad C2:Metromix 200 in 36-well trays and stratified at 4 °C for 7 d in a cold-room. Seedlings were then germinated in controlled-environment chambers at 20 °C with 70% humidity, on a 16h light/8h dark cycle and a daily watering schedule. Plants were thinned and randomized into treatment flats by day 7 of growth. On day 7, flats were transferred into one of eight treatment conditions. (i) Control plants remained in long-day conditions in a 16h light/8h dark chamber. (ii) Short-day plants experienced a 12h light/12h dark cycle. (iii) High temperature plants were exposed to 28 °C with 30% humidity on a 16h light/8h dark cycle. (iv) High water plants were watered heavily each day in trays lacking drainage and with lids on to retain humidity, while (v) low water plants were watered every 3 d with drainage and without lids. (vi) High temperature, low water plants were transferred into the 28 °C chamber as in (iii) and watered every 3 d. (vii) Cold- and (viii) heat shock-treated plants were treated as (i) control plants until 3h before harvest, when cold shock plants were placed on ice in a 4 °C chamber and heat shock plants were placed in a 37 °C incubator. To standardize the growth stage measured for different accessions, individual plants were harvested at the eight-leaf stage: the above-ground tissue was flash-frozen in liquid nitrogen, ground using a mortar and pestle, and stored at –80 °C.

### RNA extraction and qRT–PCR

To provide sufficient tissue for RNA extraction, two plants from each accession in each treatment were pooled to create one biological replicate. Three biological replicates per treatment by accession combination were processed in randomized blocks for the RNA extraction, reverse transcription, and quantitative reverse transcription–PCR (qRT–PCR) steps. RNA was extracted using a Sigma Spectrum Plant Total RNA Kit protocol with the on-column DNase I digestion procedure, and tested for quality via nanodrop. A 1 µg aliquot of RNA per 20 µl reaction was reverse transcribed per biological replicate using random hexamers (1mM, IDT), dNTPs (2mM, TAKARA), and 200U of buffered M-MuLV reverse transcriptase. Primers for qRT-PCR (Supplementary Table S1 at *JXB* online) had an *R* ^2^ of ≥0.99 and an efficiency of 1.9–2.1 on a 3-fold dilution series. Primers were specific to a particular *R*-gene within *A. thaliana* based on Primer-BLAST, and did not non-specifically amplify DNA from the 10 most common bacterial endophytes found in *A. thaliana* leaves. Three reference primers, for PP2A, helicase, and basic helix–loop–helix (bHLH), were used to normalize RNA levels between samples because these genes are stably expressed in a suite of abiotic conditions (Czechowski *et al.*, 2005).

### Real-time qRT–PCR conditions

qRT–PCRs (20 µl) were performed in 384-well plates with an ABI 7900HT sequence detection system (Applied Biosystems) using SYBR Green for dsDNA synthesis detection, and ROX red to control for differences in fluorescence between samples. Three technical replicates were run per biological sample with the following thermal profile: 95 °C for 60s, 40 cycles of 95 °C for 30s, 51 °C for 30s, and 68 °C for 40s, followed by 95 °C for 15s. Dissociation curves of the amplicons were determined by heating from 60 °C to 95 °C with a 2% ramp rate.

### qRT–PCR data cleanup and analysis

Data cleanup and analysis proceeded as described by Rieu and Powers (2009) and Vandesompele *et al.* (2002). In addition, to reduce non-specific amplification, wells with amplicon melting temperatures below those corresponding to the product of interest were removed from the analysis. The dissociation curves for each reaction were plotted and those with irregular features were removed. After data cleanup, efficiencies (E) and Ct values were calculated from the clipped qRT–PCR output using the qpcR R package and subtracting baseline data from cycles 1–8 of the PCR (Spiess *et al.*, 2008). Relative quantities (RQS) of the amplicon in the starting sample were calculated according to the formula: RQ=1/(E^Ct^). RQs were normalized by dividing the RQs by the geometric mean of the RQs for the three reference genes. Log base 2 of the normalized RQ values, which follow a normal distribution, were used as dependent variables in a linear model (LM). LM fits and residuals were robust to the exclusion of the two genes with known common insertion–deletion polymorphisms (data not shown).

To determine which variables had large effects on *R*-gene expression (accession, treatment, or accession×treatment), an LM was constructed relating *R*-gene expression to treatment, accession, and their interaction, with *R*-gene and interaction terms that included *R*-gene treated as random effects. Variance components were estimated by REML, and 95% confidence intervals for the proportions of variance explained by the random effects were estimated by performing 1000 parametric bootstraps. To extend the model to all *R*-genes, models treated *R*-gene and interaction terms including *R*-gene as random effects. To correlate *R*-gene expression with short-term environment, an LM was constructed relating *R*-gene expression to treatment, with *R*-gene, accession, and interaction terms that included *R*-gene treated as random effects, and omitting an interaction term between accession and treatment. All 12 accessions were considered in analyses involving treatment alone.

### Expression meta-study

One limitation of the qPCR study was that it could not test whether the *R*-gene response to each perturbation was unusual compared with the response of the remainder of the transcriptome. Thus, to place the qRT–PCR results into a genomic context, a meta-study of publicly available data from the EMBL-EBI Gene Expression Atlas was conducted. Expression data were collected for all available *R*-genes, and for two additional gene sets of equivalent number, generating a set of 433 genes (Supplementary Table S2 at *JXB* online). First, a control gene set was chosen to mimic the positions and distances among *R*-genes to control for co-regulation of clustered genes. Secondly, a stress response gene set was chosen randomly from genes with a Gene Ontology (GO) annotation of response to stress (GO:0006950). Most experiments included in the metastudy considered only a single accession (typically Col-0).

Expression data were collected for the 15 perturbations described below (Supplementary Table S3 at *JXB* online). The *R*-gene expression response was compared with the control gene expression response to test if the *R*-gene expression response is unusual; if so, then *R*-gene expression may be shaped by costs and benefits that the rest of the transcriptome does not experience. The *R*-gene expression response was also compared with the stress gene response to determine if the *R*-gene expression response was unusual compared with stress response genes. Eight conditions involved perturbations with pathogen or pathogen-associated molecular patterns and were expected to up-regulate *R*-genes. Ozone exposure was expected to up-regulate *R*-genes, as ozone wounds plant tissue and might allow easier infection (Sandermann, 2000). Exposure to salicylic acid (SA) was expected to up-regulate *R*-genes, as SA induces the secondary immune response and can lead to systemic acquired resistance to pathogens. Four additional perturbation treatments were considered: two changes in temperature (from 20 °C to 4 °C or 37 °C), drought stress, and 30–120min of intense light after low light. *R*-genes should be up-regulated in changing environmental conditions if changing environments increased the chance of microbiome invasion by pathogens. On the other hand, if *R*-gene expression tracked the risk of infection, then *R*-genes should be up-regulated in cold and wet environments and down-regulated in hot and dry environments (Holub *et al.*, 1994; Szittya *et al.*, 2003).

For each gene, expression was scored as up-regulated, down-regulated, or non-differentially expressed relative to the controls for each experimental condition. Benjamini–Hochberg-corrected χ^2^ tests of numbers of *R*-genes up-regulated, down-regulated, or non-differentially expressed were conducted to assess if *R*-genes responded significantly differently to environmental perturbations compared with control or stress response genes.

### Resistance determination

A second limitation of the qPCR study was that it did not link an expression response upon treatment with a phenotypic response, such as a change in resistance. To determine if short-term environmental changes could influence or ‘prime’ disease resistance, Col-0 plants were grown as in the qRT–PCR study, transferred just prior to the 21 d stage into one of three treatments, then immediately post-treatment, at the 21 d stage, infected with one of two pathovars of *P. syringae*, at an OD_600_ of 0.002 by blunt-end syringe inoculation (Korves and Bergelson, 2003). Mock-infected plants were inoculated with the buffer 10mM MgSO_4_. After inoculation, plants were kept at high humidity under plastic domes for 3 d to facilitate infection. Plant resistance was measured as the log of the number of colony-forming units (CFU) in the infected leaf 3 d post-infection). The CFU were measured by plating dilutions of ground leaf punches on Luria–Bertani plates with blinded colony counting. A total of 20–26 replicates per combination of pathovar and treatment were used (Supplementary Data set S2 available at *JXB* online).

Control plants and cold-shocked plants were treated as in the qRT–PCR study. The positive control, BTH (butylated hydroxytoluene)-treated plants, were treated as control plants until 24h before harvest, then sprayed with 100 µM BTH, a SA analog that both induces *R*-gene expression and increases pathogen resistance (Wang *et al.*, 2006). Two pairs of DC3000 strains were used: one pair with and without the RPS2-recognized avirulence gene *avrRpt2* (Guttman and Greenberg, 2001), and one pair with and without the RPS5-recognized avirulence gene *avrPphB* (Karasov *et al.*, 2014). The DC3000 strains contained an empty version of the different vectors used to introduce the two avirulence genes.

### Gene expression clines: experimental strategy and overview

Clinality in a trait, or a gradient in that trait that follows an environmental gradient, is often the first line of evidence for local adaptation in that trait (Clausen *et al.*, 1940). Drawing on data from the qPCR experiment described above, clinality in *R*-gene basal expression and *R*-gene expression plasticity was explored in a worldwide set of six accessions (hereafter the ‘Clark worldwide set’) argued to capture most of the species’ genetic diversity without strong effects of population structure (Clark *et al.*, 2007). One limitation of this analysis was that it could not test whether clinality in *R*-gene expression was unusual compared with the clinality in the remainder of the transcriptome. To test this, the set of accessions of Gan *et al.* (2011) (hereafter the ‘Gan worldwide set’) was re-analyzed to provide a neutral expectation for expression clinality across the transcriptome for a worldwide set of accessions. A second limitation of this analysis was that the worldwide set was not distributed along a traditional cline. Having found weak evidence for clinality in *R*-gene expression in the worldwide data set, two additional sets of accessions collected from latitudinal clines at spatial scales of hundreds of kilometers were examined for further evidence of *R*-gene expression clinality. One of these latitudinal clines consisted of the described qPCR data for five accessions in a Midwestern US latitudinal cline (hereafter the ‘Midwestern set’). The second latitudinal cline consisted of RNA-seq data of 144 accessions (hereafter the ‘Swedish set’) from two Swedish populations grown in a common growth chamber condition (Dubin *et al.*, 2015). To test if *R*-gene expression clinality was unusual, the Swedish set was used to provide a neutral expectation for expression clinality across the transcriptome.

### Clark worldwide set analyses

Natural variation in gene expression was first explored in the worldwide set to examine *R*-gene expression patterns across the entire species range. To correlate *R*-gene expression with historical climates from which accessions derived, four climate variables from Hancock *et al.* (2011) were considered: latitude, minimum temperature in the coldest month, precipitation in the driest month, and temperature seasonality (maximum temperature in the warmest month–minimum temperature in the coldest month). With the exception of latitude, these climate variables were highly correlated: more temperate climates had both warmer winters and were drier. Two of 12 accessions were eliminated from climate analysis: Col-0 and Tsu-1, accessions with unknown locations of origin (Anastasio *et al.*, 2011).

To explore the association between basal expression and climate, LMs were first constructed relating *R*-gene expression in short days and in long days to accession, with *R*-gene treated as a random effect. Then, the model estimate of *R*-gene expression for each accession was regressed against each of four climate variables, and model slopes were determined. Basal expression in short-day conditions is more biologically relevant than that under long-day conditions for *A. thaliana* collected at the eight-leaf stage, as this day length corresponds to spring growth conditions. *P*-values of the model slopes were determined to identify significant effects of climate on basal *R*-gene expression. For each treatment, model slope *P*-values were Bonferroni corrected for the number of climate variables compared.

To explore the interaction between accession and treatment, LMs were constructed relating *R*-gene expression and accession, in each of the seven treatments separately, with *R*-gene treated as a random effect. Average *R*-gene expression plasticity, or the fold change in expression upon perturbation was then determined by calculating the differences between the model estimates of average *R*-gene expression levels after each treatment and ‘control’ levels for plants that remained in long days. This method corrected for primer bias between accessions. To explore how historic climate influenced plasticity in *R*-gene expression upon environmental perturbation, LMs of average *R*-gene plasticity against four climate variables were constructed and model slopes were determined. For each treatment, *P*-values were Bonferroni corrected for the number of climate variables compared.

### Gan worldwide set analyses

To test if the temperature seasonality cline in *R*-gene expression was unusual, RNA-seq data from 14 of 19 accessions in the study of Gan *et al.* (2011) with climate data from Hancock *et al.* (2011) were used, and Col-0, Hi-0, Po-0, Oy-0, and Tsu-0 were excluded. To explore the relationship between temperature seasonality and average *R*-gene expression, an LM relating the normally distributed, average expression of all *R*-genes against temperature seasonality was constructed. Secondly, a null distribution of *t*-values of 1000 LMs of the average expression of 150 randomly sampled genes against temperature seasonality was constructed. A ‘stress response gene’ distribution was also created by establishing sets of 150 genes with a GO annotation of response to stress (GO:0006950). The *t*-value from the LM of *R*-gene expression was compared with these two null distributions to determine if the clinality of *R*-gene expression associated with temperature seasonality was likely to have occurred by chance, or unusual in comparison with other stress response genes.

### Midwestern set analysis

The Midwestern set was used to explore *R*-gene expression clinality along a latitudinal cline. In the Midwestern US, *A. thaliana* populations exhibit local isolation by distance that extends hundreds of kilometers (Platt *et al.*, 2010), and natural variation in *R*-genes has previously been found to be segregating in the Midwestern US (Karasov *et al.*, 2014). The interaction between basal expression and climate in the Midwestern set was explored as described for the Clark worldwide set.

### Swedish set analyses

The Swedish set was used to explore *R*-gene expression clinality in an independent latitudinal cline. This data set included RNA-seq data from 144 accessions with climate data from Hancock *et al.* (2011) from two Swedish populations grown in a common growth chamber condition, and is described further in Dubin *et al.* (2015). Previous work has demonstrated that these Swedish accessions should be treated as two distinct populations (Huber *et al.*, 2014). Thus, three analyses were conducted. First, an LM of average *R*-gene expression against latitude, as in the qPCR data, was constructed. Secondly, sets of 150 randomly chosen genes were averaged, then *t*-values of the coefficients of 1000 LMs of these sets against latitude were determined to create a null distribution of how an expression data set such as that for the *R*-genes would be expected to vary with latitude at random. The *t*-value from the LM of average *R*-gene expression was compared with this null distribution to determine if the latitudinal correlation of *R*-gene expression was likely to have occurred by chance. To control for population structure or effects of genome size that might differ between northern and southern Sweden (Long *et al.*, 2013), the expression of each *R*-gene and each control gene was tested within each population to determine whether expression was significantly higher in the northern or southern Swedish population.

## Results

### Current and historic environment interact to influence *R*-gene expression levels

The effects of the eight abiotic treatments (seven perturbations plus control) and 12 accessions on *R*-gene expression were determined using an LM of normalized expression data (Tables 2, 3). The random effect of gene explained 55% of the variation in R-gene expression, and 28% of the variation was explained by an interaction between gene and accession (Table 3). Model comparisons using the Akaike information criterion (AIC), log likelihood, and χ^2^ values indicated that the two-way interaction between accession and treatment was highly significant. This interaction had a stronger effect on *R*-gene expression than the effect of accession or treatment alone (mean squares=22.9, 12.9, 16.9), and abiotic treatment had a stronger effect on *R*-gene expression than the effect of accession alone (mean squares=16.9, 12.9). The effects of treatment, accession, and their interaction in this data set were thus all further explored; the effects of accession and its interaction with treatment were explored as functions of historic climate.

**Table 2.** Fixed effects for a mixed effect linear model fitted to the log_2_ of the normalized relative quantities of RNA, with treatment, accession, and their interaction modeled as fixed effects, and both *R*-gene and two-way interaction including *R*-gene modeled as random intercepts

Fixed effects	df	Sum sq.	Mean sq.	*F*
Accession	11	144.92	13.18	3.93
Treatment	7	213.0	30.43	9.08
Accession×Treatment	46	1056.8	22.98	6.86

**Table 3.** Random effects for the mixed effect linear model described in Table 2

Random effects	Type	Variance	Variance component (confidence interval)
Gene×Accession	(Intercept)	5.72	0.282 (0.165–0.443)
Gene	(Intercept)	11.21	0.553 (0.319–0.746)
Residual		3.35	0.165 (0.0978–0.248)

**Fig. 1.**
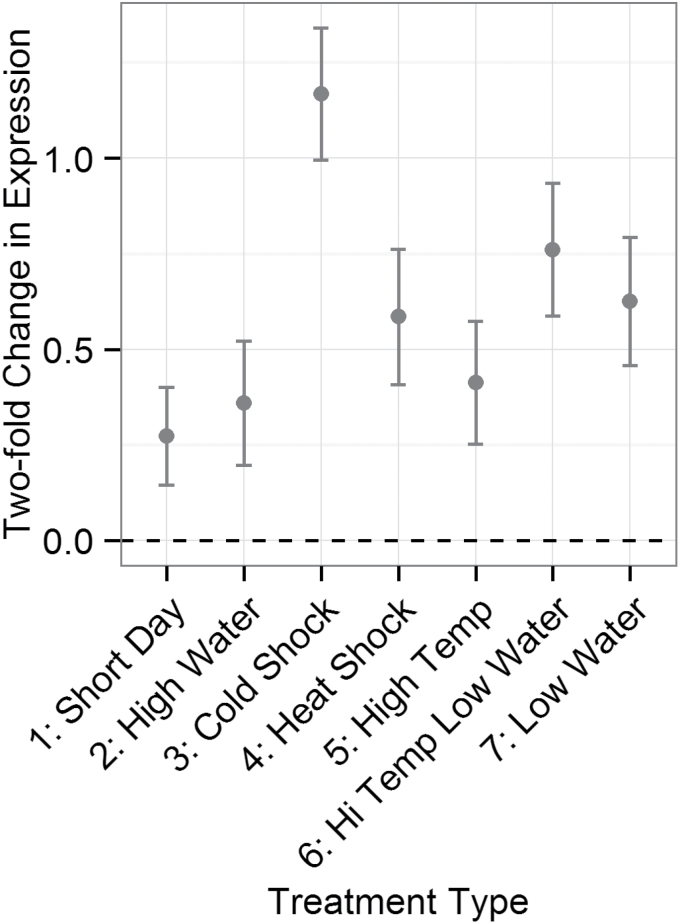
Coefficients of a linear model of *R*-gene expression against treatment for 13 *R*-genes in 12 accessions in *Arabidopsis thaliana*. *R*-genes and all interactions with *R*-genes were included as random effects in the model. The *y*-axis corresponds to a log_2_ fold change in expression for each treatment relative to the control treatment.

The coefficients of an LM of *R*-gene expression as a function of short-term environmental perturbation, or treatment, were determined. *R*-genes were significantly up-regulated in all treatments (Fig. 1). The strongest response, an average 2.5-fold up-regulation of *R*-genes, occurred after 3h of cold shock (Fig. 1). A more modest response (1.6-fold) was seen for the week-long temperature and water treatments, and for the 3h heat shock.

### *R*-gene expression responses are distinct from non-*R*-gene expression responses

The responses of *R*-genes and non-*R*-genes were compared using 15 published conditions that involved environmental perturbation followed by expression measurements hours to days later (Table 4). Significant differences in the response of *R*- and control gene sets were observed for infection with two pathogens, *Hyaloperonospora parasitica* and powdery mildew, and for treatment with SA (Table 4). Up-regulation of more *R*-genes relative to control genes drove the difference in expression (2×2 χ^2^ tests). *R*-genes were up-regulated on average in 10 of 15 perturbations (Table 4). For 14 of 15 perturbations, there was more consistency in the differentially expressed *R*-genes towards up-regulation or down-regulation than in control genes, while control genes were more evenly split between these groups.

**Table 4.** The number of *R-,* control, and stress response genes differentially expressed after 15 environmental perturbations. Datasets used can be found in the Supplementary Information.

Treatment and gene set	Up-regulated	No change	Down-regulated	Sets compared	χ^2^ value	*P*-value
*Blumeria graminis* versus uninfected						
* R*-genes	6	5	5	*R* versus control	2.97	0.23
Control	5	15	6	*R* versus stress	1.48	0.48
Stress response	11	7	17			
*Hyaloperonospora parasitica arabidopsis Noco2* versus uninfected						
* R*-genes	33	25	2	*R* versus control	18.7	8.7E-05*
Control	12	28	14	*R* versus stress	8.65	**0.013**
Stress response	52	26	17			
Powdery mildew (*Erisyphe cichoracearum*) versus uninfected						
* R*-genes	35	11	2	*R* versus vontrol	18.6	9.2E-05*
Control	5	18	1	*R* versus stress	14.7	6.3E-04*
Stress response	22	31	8			
*Pseudomonas syringae* pv. DC3000 with *avrRpm1*versus uninfected						
* R*-genes	8	3	3	*R* versus control	2.84	0.24
Control	6	4	0	*R* versus stress	3.28	0.19
Stress response	10	15	7			
*Pseudomonas syringae* pv. *Maculicola* with *AvrRpt2* versus mock inoculated						
* R*-genes	36	16	9	*R* versus control	4.73	0.094
Control	24	16	17	*R* versus stress	12.1	2.3E-03*
Stress response	45	10	34			
*Pseudomonas syringae* pv. tomato with *HopZ1a* versus mock inoculated						
* R*-genes	26	15	11	*R* versus control	0.49	0.78
Control	16	8	9	*R* versus Stress	1.72	0.42
Stress response	40	20	27			
*Pseudomonas syringae* pv. tomato versus mock inoculated						
* R*-genes	2	10	14	*R* versus control	6.37	**0.041**
Control	4	6	2	*R* versus stress	10.1	**6.3E-03**
Stress response	6	37	10			
flg22 peptide versus water						
* R*-genes	4	4	0	*R* versus control	3.05	0.22
Control	8	5	5	*R* versus stress	7.35	**0.03**
Stress response	5	6	13			
Salicyclic acid versus Silwet, <24h after treatment						
* R*-genes	47	18	4	*R* versus control	11.8	2.8E-03*
Control	14	17	8	*R* versus stress	39.5	2.6E-09*
Stress response	33	76	31			
Salicyclic acid versus Silwet, >24h after treatment						
* R*-genes	0	18	21	*R* versus control	1.24	0.54
Control	0	6	3	*R* versus stress	9.53	**0.01**
Stress response	3	32	11			
0.5h or 2h excess light versus low light						
* R*-genes	5	33	34	*R* versus control	9.22	**0.010**
Control	11	29	13	*R* versus stress	10.6	**5.1E-03**
Stress response	35	57	48			
37 °C versus 20 °C						
* R*-genes	7	23	22	*R* versus control	3.09	0.21
Control	11	15	13	*R* versus stress	1.19	0.55
Stress response	18	39	32			
4 °C versus 20 °C						
* R*-genes	13	45	24	*R* versus control	10.3	**5.8E-03**
Control	15	35	16	*R* versus stress	24.8	4.0E-06*
Stress response	56	65	36			
Drought versus untreated						
* R*-genes	18	30	4	*R* versus control	3.50	0.17
Control	8	23	7	*R* versus stress	8.77	**0.012**
Stress response	30	36	25			
Ozone versus control						
* R*-genes	8	6	3	*R* versus control	2.43	0.30
Control	2	6	3	*R* versus stress	0.69	0.71
Stress response	17	9	9			

*P*-values in bold are significant correlations, and asterisks represent significant correlations after correction for multiple testing.

To determine if the *R*-gene response was typical for stress response genes, the same comparisons were made with stress response genes. Significant differences in the response of *R*-gene and stress response sets were observed for four treatments: infection with two pathogens, powdery mildew and *P. syringae* pv. *maculicola* with *AvrRpt2*, treatment with SA, and cold treatment (Table 4). Again, the expression response of *R*-genes to treatment was more consistent than the response of stress-related genes, with stress response genes more evenly split in terms of the number of genes up- and down-regulated in each treatment.

### Environment prior to infection can prime disease resistance

**Fig. 2.**
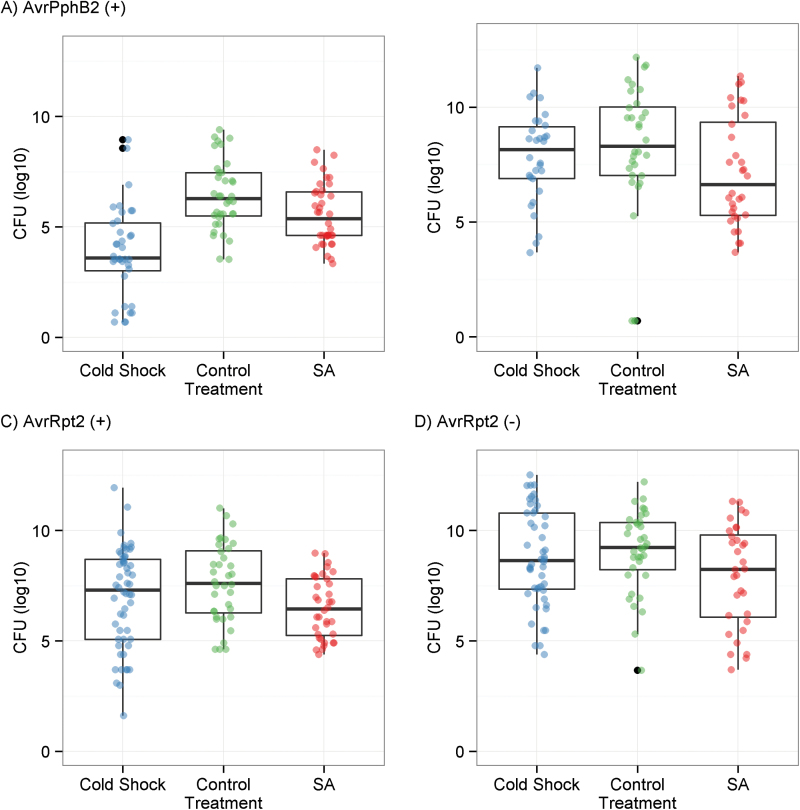
Bacterial titers measured 3 d post-infection for *Arabidopsis thaliana* plants exposed to three treatments then infected by two pairs of *Pseudomonas syringae* strains. Data are overlaid as jittered points. (A) Infection with *P. syringae* pv. *AvrPphB2*; (B) infection with *P. syringae* (–) *AvrPphB2*; (C) infection with *P. syringae* pv. *AvrRpt2*; (D) infection with *P. syringae* (–) *AvrRpt2*. (This figure is available in colour at *JXB* online.)

To explore the effects of environmental perturbation on pathogen resistance, plants exposed to cold shock, the perturbation with the strongest *R*-gene response, were infected with two pairs of *P. syringae* pathovars with and without the avirulence genes *AvrPphB2* and *AvrRpt2*, cognate avirulence genes of the *R*-genes *Rps5* and *Rps2.* The positive control, an SA analog, increased resistance to all four pathogen strains (Fig. 2). Cold shock did not lead to an increase in resistance to strains lacking avirulence genes nor did it alter specific pathogen resistance to *P. syringae* pv. *AvrRpt2* (Fig. 2). In contrast, cold shock led to a significant increase in resistance to *P. syringae* pv. *AvrPphB2* (Fig. 2). The increase in resistance to *P. syringae* pv. *AvrPphB2* was striking, a 340-fold decrease in CFU compared with an average 9-fold decrease in CFU with SA treatment.

### Basal *R*-gene expression and *R*-gene expression plasticity vary clinally at a worldwide scale

**Fig. 3.**
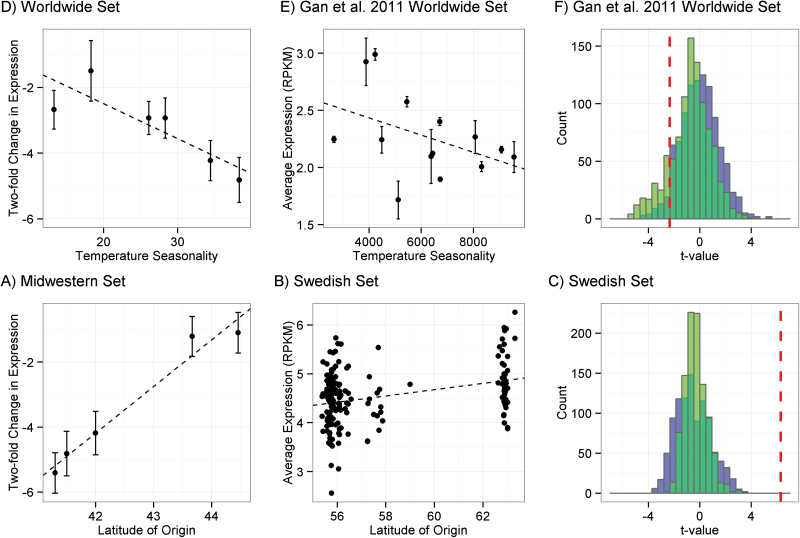
Clinal variation in basal *R*-gene expression in *Arabidopsis thaliana*. Dashed lines are regression lines. (A, D) The *y*-axes corresponds to the log_2_ basal expression for each accession in short-day conditions. (A) Expression variation of 13 *R*-genes in accessions from the Clark worldwide set against temperature seasonality at the accession’s location of origin. (B) Average expression of 150 *R*-genes in accessions from the Gan worldwide set against temperature seasonality at the accession’s location of origin. (C) The null distributions of *t*-values of correlations with temperature seasonality for 1000 sets of 150 randomly sampled gene expression profiles from the Gan worldwide set. Null distributions were drawn from all genes (blue) and stress response annotated genes (green). The dashed vertical line is the *t*-value of the correlation from (B). (D) Expression variation of 13 *R*-genes in accessions from the Midwestern set against latitude of origin. (E) Average expression of 150 *R*-genes in accessions from the Swedish set against latitude of origin. (F) The null distributions of *t*-values of correlations with temperature seasonality for 1000 sets of 150 randomly sampled gene expression profiles for the Swedish set. Null distributions were drawn from all genes (blue) and stress response annotated genes (green). The dashed vertical line is the *t*-value of the correlation from (E).

To explore the effect of historic environment on *R*-gene expression in the Clark worldwide set, basal *R*-gene expression in short days was regressed against four climate variables. Basal *R*-gene expression in short days was correlated with temperature seasonality (*R* ^2^=0.73, *P*=0.031), although this correlation was not significant after a Bonferroni correction. Here, higher basal expression of *R*-genes was associated with lower temperature seasonality, or a more temperate winter climate (Fig. 3A). In the Gan worldwide data set, average *R*-gene expression also varied significantly with temperature seasonality (*R* ^2^=0.185, *t*= –2.349, *P*=0.022; Fig. 3B). However, null distributions of averaged expression of sets of randomly sampled genes and stress response genes both overlapped this value, with 4.8% of the null distribution and 16% of the stress response gene distribution as extreme as or more extreme than the relationship between *R*-genes and temperature seasonality (Fig. 3C).

**Fig. 4.**
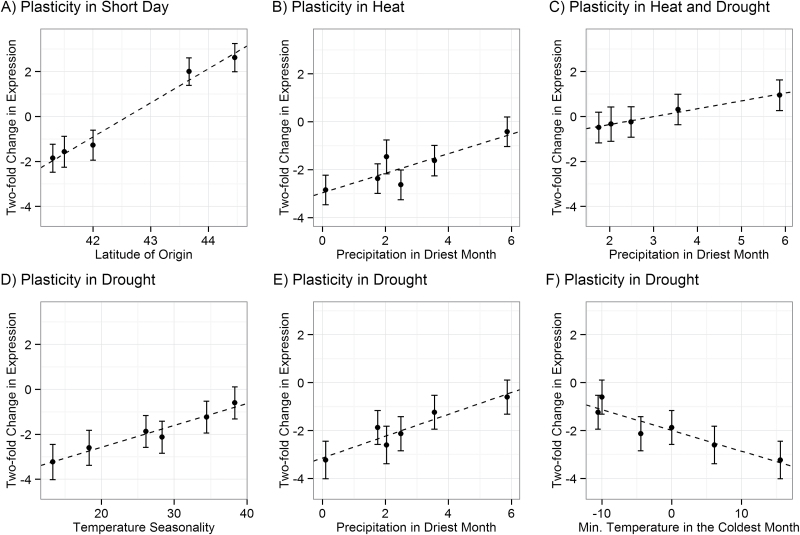
Clinal variation in resistance (*R*-) gene expression plasticity in *Arabidopsis thaliana.* The *x*-axes correspond to historical climate variables. The *y*-axes correspond to a log_2_ fold change in expression for each treatment relative to the control treatment. Dashed lines are the regression lines. (A) Plasticity in *R*-gene expression upon change to short days correlates with latitude of origin for accessions in the Midwestern set. (B, C) Plasticity in *R*-gene expression after heat, and heat and drought stress correlates with precipitation differences at the accession’s location of origin in the Clark worldwide set. (D–F) Plasticity in *R*-gene expression after drought stress correlates with precipitation differences and temperature differences at the accession’s location of origin in the Clark worldwide set.

To explore the interaction between accession and treatment in the Clark worldwide set, the relationships between historical climate variables and *R*-gene expression plasticity in seven environmental perturbations in the qPCR set were examined. There were no significant clines in *R*-gene expression when plants were perturbed with short days, high water, cold shock, or heat shock. Expression changes after mild drought were significantly correlated with minimum temperature in the coldest month and temperature seasonality (Table 5). In particular, accessions from temperate climates, with milder winters, tended to down-regulate *R*-genes significantly more than accessions from more continental climates, with colder winters (Fig. 4D, E). Expression changes in response to high temperature, mild drought, or both were correlated with precipitation in the driest month (Table 5). Accessions from dry climates down-regulate *R*-genes significantly more than accessions from wetter climates in increased heat and aridity (Fig. 4B, C, E).

**Table 5.** Correlations between average *R*-gene expression and four climate variables. Significant correlations of both basal *R*-gene expression and *R*-gene expression plasticity after environmental perturbation are shown. *These clines are displayed in Figures 3a, 3d, and 4.*

Accessions	Climate variable	Treatment	Slope	*R* ^2^	Adjusted *R* ^2^	*F*	*P*-value
*Basal expression*							
Midwest	Latitude	Short day	1.435	0.964	0.953	81.2	0.003*
Worldwide	Temperature seasonality	Short day	–0.107	0.728	0.661	10.75	**0.031**
*Expression plasticity*		*Fold change in response to:*					
Midwest	Latitude	Short day	1.516	0.981	0.975	157.7	0.001*
Worldwide	Temperature seasonality	Low water	0.097	0.954	0.943	83.5	<0.001*
Worldwide	Minimum temperature in the coldest month	Low water	–0.087	0.864	0.830	25.4	0.007*
Worldwide	Precipitation in the driest month	Low water	0.456	0.887	0.858	31.2	0.005*
Worldwide	Precipitation in the driest month	High temperature and low water	0.350	0.983	0.977	169.3	<0.001*
Worldwide	Precipitation in the driest month	High temperature	0.408	0.764	0.705	12.9	**0.023**

*P*-values in bold are significant correlations, and asterisks represent significant correlations after corrections for multiple testing to four climate variables.

### Basal *R*-gene expression increases with latitude in two latitudinal clines

In the Midwestern set, there was a significant correlation between basal *R*-gene expression in short days and latitude of origin (*R* ^2^=0.96, *P*=0.003), with accessions from higher latitudes exhibiting higher basal expression levels (Fig. 3D). Other climate variables were uncorrelated with basal *R*-gene expression in short-day conditions. In the Midwestern set, the responsiveness of *R*-genes to a change to short days was also significantly correlated with latitude of origin (*R* ^2^=0.97, *P*=0.002; Fig. 4A). Other climate variables were uncorrelated with changes in expression upon a transition to short days.

In the Swedish set, an LM indicated that average *R*-gene expression was significantly higher in the northern than in the southern Swedish population (*R* ^2^=0.116, *t*=6.29, *P*=2.9e-07; Fig. 3E). The *t*-value of the coefficient of this relationship was higher than the entire null distribution of sets of randomly sampled genes and stress response genes (Fig. 3F). Higher gene expression in northern Sweden was not seen on a gene-by-gene basis: control genes had roughly equal proportions of genes, with higher gene expression in northern or with higher gene expression in southern Sweden, while the vast majority of differentially expressed *R*-genes were expressed at higher levels in the northern population (Supplementary Fig. S1 at *JXB* online).

## Discussion

*R*-genes were up-regulated in response to all abiotic treatments in this expression study, with the largest up-regulation occurring after cold shock (Fig. 1). Neither the duration nor the type of short-term treatment affected the direction of the average *R*-gene response. The meta-study reveals that not only are *R*-genes typically up-regulated in response to perturbations but, for many perturbation responses, the *R*-gene expression response is more consistent and biased towards up-regulation than the response of control or stress response genes (Table 4). Though other stress response gene sets or other components of the defense response might also show similar biases in expression response, extensive additional work would be required to explore adaptive responses of other components of the plant response to stress and the plant response to pathogens. The bias towards *R*-gene up-regulation is not consistent with the idea that plants track the weather, increasing or decreasing *R*-gene expression in response to how favorable the environment is for pathogen growth. Instead, this suggests that changes in the environment *per se* increase risk of infection.

It is possible that the *R*-gene expression responses are adaptive because species are more likely to invade a community successfully after disruption (Shea and Chesson, 2002). For bacterial communities in particular, both biotic and abiotic environmental factors have been shown to alter the plant microbiome (Kadivar and Stapleton, 2003; Suda *et al.*, 2009; Yutthammo *et al.*, 2010; Ikeda *et al.*, 2011), and changes in abiotic conditions modulate the risk of infection by both latent and novel pathogens (Szittya *et al.*, 2003; Yi *et al.*, 2004; Monteiro *et al.*, 2012). While details of the relationship between microbiome structure and plant resistance are yet to be understood, many microbial species take part: bacteria from Proteobacteria, Firmicutes, and Actinobacteria are known to contribute to the suppression of fungal and bacterial pathogens (Innerebner *et al.*, 2011; Mendes *et al.*, 2011), and axenic plants are known to be especially susceptible to infection by pathogens (Innerebner *et al.*, 2011). The significant increase in disease resistance after cold shock demonstrates that the environment can prime disease resistance (Fig. 2). Thus, the consistent up-regulation of *R*-genes in response to environmental change could be a preparatory response to conditions that alter the probability of invasion by pathogens.

Clinal interactions between *R*-gene expression plasticity and historic environment support fine-scale genetic tinkering to adapt *R*-gene expression levels to the environment (Table 5). The significant interaction between accession and abiotic treatment suggests that selection has shaped the plasticity of *R*-gene expression as a function of the genotype collected from different historical climates (Table 2). The Clark worldwide set has an 8-fold difference in basal *R*-gene expression across the range of temperature seasonality in data sets (Fig. 3A). This expression difference was recapitulated for all *R*-genes in the Gan worldwide set (Fig. 3B). However, both null distributions showed more extreme clinality than *R*-genes (Fig. 3C); thus, this expression difference could have arisen through random drift in *R*-gene expression levels between populations. Alternatively, permissive growth chamber conditions may fail to mimic the environment in which *R*-gene expression is under selection. In other words, phenotypic consequences of *R*-gene expression in different environments could affect *R*-gene expression levels in the conditions tested here. In this vein, a 4-fold difference in average *R*-gene expression was observed in response to drought stress across the climatic range of temperatures and aridity in the Clark worldwide set (Fig. 4D, E, F). Four of these plasticity clines were significant after a Bonferroni correction for multiple climate variables, while the basal expression cline was not significant after a Bonferroni correction (Table 5). Though basal *R*-gene expression levels were higher in accessions from more temperate climates, accessions from these climates down-regulated *R*-genes in drought to a much greater degree than accessions from colder, wetter climates (Figs 3, 4). Similarly, accessions from dry climates down-regulated *R*-genes in response to increased heat and aridity more than accessions from wet climates (Fig. 4B, C). The consistency of the accession-specific expression responses to heat, drought, and heat plus drought strengthens support for a relationship between *R*-gene expression plasticity and aridity of the historic climate (Table 5).

The observed expression responses are inconsistent with the idea that plants track the weather, increasing or decreasing *R*-gene expression in direct response to how favorable the environment is for pathogen growth. Instead, they point to the possibility that changes in the environment *per se* act to promote infection by pathogenic species. It is known that rare environmental disruptions are more likely to promote invasion than common environmental disruptions (Sher and Hyatt, 1999). Thus, identical weather events may differentially affect accessions depending on their historical climates. For example, wetter climates typically experience fewer droughts, and this rare disruption may select for relatively greater up-regulation of *R*-genes in plants from wetter climates. Thus, *R*-gene up-regulation may be a preparatory response to an increased likelihood of microbiome invasion. These interactions between weather and climate generally support the idea that *R*-gene expression changes are a mechanism to defend against changes to the microbial community; *R*-genes may be induced to prevent plant microbiome invasion when an unusual perturbation occurs.

Historical climate appears particularly to influence basal *R*-gene expression across latitudinal clines at medium spatial scales. The Midwestern set shows a strong trend in basal *R*-gene expression, with the northernmost accession expressing *R*-genes 20-fold more strongly than the southernmost accession (Fig. 3D). In the Swedish set, latitude explained a small, but significant, fraction of the variation in average *R*-gene expression. Moreover, this distribution of *R*-gene expression values is extremely unlikely to have arisen by chance or by drift in gene expression between populations (Fig. 3F); thus, *R*-gene expression is probably under differential selection between the two Swedish populations. Gene by gene, *R*-gene expression was consistently higher in the northern Swedish population than in the southern Swedish population: 26 of 29 *R*-genes that were significantly differentially expressed between populations after a Bonferroni correction were expressed at a higher level in northern Sweden (Supplementary Fig. S1 at *JXB* online). These clinal patterns are consistent with the resource allocation hypothesis, which suggests that defense should increase as plant tissue becomes more difficult to acquire (Coley *et al.*, 1985), such as in locations with shorter day lengths. Of course, many other factors, such as pathogen distributions and abundance, may select on *R*-gene expression patterns and explain the residual variation in *R*-gene expression. Unfortunately, little is known about the distribution of *A. thaliana* pathogens through time and space.

In conclusion, inducible defenses are a cost-saving strategy, and are theorized to occur only in environments where they confer a fitness benefit to the individual (Cipollini, 2008). *R*-genes initiate one such inducible defense, and their up-regulation can benefit plant fitness through increased pathogen resistance but can be costly in the absence of pathogens. *R*-gene family expression is distinct from an average transcriptome response after biotic perturbations and across a latitudinal cline, supporting the idea that *R*-gene expression is shaped by these additional selective forces. *R*-genes are induced when the biotic or abiotic environment is perturbed, especially when that perturbation is unusual given the historical climate. Basal *R*-gene expression increases at higher latitudes, consistent with the resource allocation hypothesis. Fine-scale genetic tinkering for differential expression responses to climate and weather perturbations may help explain the atypically high natural variation in *R*-gene expression.

## Supplementary data

Supplementary data are available at *JXB* online.

Table S1, Resistance (*R*) genes and primers used in the expression study.

Table S2. Genes considered in the meta-study and the gene set they were in.

Table S3. Treatments considered in the meta-study and the studies from which they were derived.

Figure S1. Clinal variation in basal expression with latitude or population in the Swedish RNA-seq data set.

Data set S1. Expression data set for the qRT–PCR study of *R*-gene expression.

Data set S2. Data set of bacterial titers shown in Fig. 2.

Supplementary Data

## Abbreviations:

GWAS: genome-wide association study
*R*-gene: resistance gene
LM: linear model
SA: salicylic acid
BTH: butylated hydroxytoluene.
