# Supplementary material for: Modulation of R-gene expression across environments
Source: J Exp Bot. 2016 Mar 15;67(7):2093–105. doi: 10.1093/jxb/erv530 (PMC4793800; doi:10.1093/jxb/erv530)
Supplement: Supplementary Data [file supp_67_7_2093__index.html]

Modulation of R-gene expression across environments — Supplementary Data 

# Modulation of *R*-gene expression across environments

## Supplementary Data

Data files

- Supplementary\_Tables\_S1\_S3\_figure\_S1.pdf - Supplementary Data
- supplementary\_dataset\_1.xlsx - Supplementary Data
- supplementary\_dataset\_2.xlsx - Supplementary Data
