# Supplementary material for: Modulation of R-gene expression across environments
Source: J Exp Bot. 2016 Mar 15;67(7):2093–105. doi: 10.1093/jxb/erv530 (PMC4793800; doi:10.1093/jxb/erv530)
Supplement: Supplementary Data [file supp_erv530_Supplementary_Tables_S1_S3_figure_S1.pdf]

Supporting Information

Article title: **Modulation of *R*-gene expression across environments**
Authors: Alice MacQueen, Joy Bergelson

The following Supplementary Material is available for this article:

Table S1 *R*-genes and primers used in qRT-PCR study, including the GenBank transcript number predicted to be recognized by the primers and the gene region (start-end) recognized by the primers. 13 *R*-genes were chosen to represent the larger set and included both singletons (seven) and genes from tandem arrays (six); genes from the TIR-NB-LRR subfamily (six), CC-NB-LRR subfamily (two), and NB-LRR subfamily (five); genes with overexpression mutants with known fitness consequences (two) and genes with known functions (three).

Gene	Name	GenBank Acc.	Subfamily	Cluster Type	Phenotype	Gene Region Amplified	Primers Used
AT1G27180		NM102480.3	TIR-NB-LRR	Tandem array		4743-4861	AGCGTGTGTGCTTCTGTAAGTTTTT<br>TTGTGGGAAACAAGAGTCTCCTCT
AT1G50180		NM103903.1	CC-NB-LRR	Singleton		1262-1405	TGCCTCCCCATGTAAAGCAATGT<br>CCACTGTCGTGCCAGCCTCT
AT1G56510	ADR2	NM104527.3	TIR-NB-LRR	Tandem array	Fitness cost (dwarfism) upon overexpression; confers resistance to four races of *Albugo candida.*	1381-1471	CACACAGAGGCTGGCACGACA<br>CCCTCCTCCCAACCATAACCATGC
AT2G14080		NM126980.2	TIR-NB-LRR	Singleton		2695-3079	TCTTGAGAGGATCACAGTGCTGGA<br>CCAGTTTTCCGCATCCGCTGAGT
AT3G07040	RPM1	NM111584.2	NB-LRR	Singleton	Fitness cost of resistance of presence; recognizes avrB and avrRpm1	2253-2372	TGACCTGATCGCAACTGCAAGCA<br>AGCTGAGATCCACGCAAACCCA
AT4G12020	MEKK4	NM001125496.1	NB-LRR	Mixed array		4733-4856	TGGAAACGGAAGAGACGGGAGC<br>TCGGAGGCATAAATCGGCGACG
AT4G26090	RPS2	NM118742.2	NB-LRR	Singleton	Recognizes avrB and avrRpt2	1854-1953	CCCGAAACTGACAACACTGATGCT<br>CAAGTCCAAGACTCTGAGAACAGGC
AT5G04720	ADR1-L2	NM120554.1	NB-LRR	Singleton		2389-2496	TGGGAAAAGGTCCAGAAGGCGG<br>GCTCGGAGGGAGAGAATCACGAA
AT5G05400		NM_120622.1	CC-NB-LRR	Singleton		1660-1778	GTCGCATGTGCCGATCCTTA<br>AACCCATCTGGAAGGCTCGTT
AT5G17880	CSA1	NM121794.2	TIR-NB-LRR	Tandem array		2469-2541	CTTGGGGAAACATGAGCCGC<br>GCATAAACGACGCACGGAGA
AT5G22690		NM122175.2	TIR-NB-LRR	Singleton		2887-2988	CAGGAGTCGGTGTACGCTTCC<br>GTTCAGTCTCAGGGCATCCACA
AT5G40060		NM123369.3	NB-LRR	Mixed array		2711-2800	CAAGGCTGACTTTTCAGACTGTGGG<br>ATATTATCTGCTGTCGCCGCCGC
AT5G44870	LAZ5	NM123855.1	TIR-NB-LRR	Tandem array		2428-2573	GCCGGGGATCTGACGAGACTT<br>ACGTCCGTTGGAACGCTCTC

Table S2 Genes considered in the metastudy and the gene set they were in.

Gene	Gene Set	Gene	Gene Set	Gene	Gene Set
AT1G01040	Stress response gene	AT1G08170	Control gene	AT4G26090	R-gene
AT1G02730	Stress response gene	AT1G08210	Control gene	AT1G10920	R-gene
AT1G04810	Stress response gene	AT1G08270	Control gene	AT1G12210	R-gene
AT1G06390	Stress response gene	AT1G08280	Control gene	AT1G12220	R-gene
AT1G08600	Stress response gene	AT1G10940	Control gene	AT1G12280	R-gene
AT1G09970	Stress response gene	AT1G13320	Control gene	AT1G12290	R-gene
AT1G11820	Stress response gene	AT1G16890	Control gene	AT1615890	R-gene
AT1G13260	Stress response gene	AT1G24140	Control gene	AT1G33560	R-gene
AT1G14755	Stress response gene	AT1G24150	Control gene	AT1G50180	R-gene
AT1G16130	Stress response gene	AT1G33580	Control gene	At1G53350	R-gene
AT1G17860	Stress response gene	AT1G34540	Control gene	At1G58390	R-gene
AT1G19380	Stress response gene	AT1G42410	Control gene	At1g58410	R-gene
AT1G21710	Stress response gene	AT1G50350	Control gene	AT1G59124	R-gene
AT1G24000	Stress response gene	AT1G52180	Control gene	At1g59218	R-gene
AT1G26700	Stress response gene	AT1G53300	Control gene	At1g59620	R-gene
AT1G28680	Stress response gene	AT1G53340	Control gene	At1g59780	R-gene
AT1G31180	Stress response gene	AT1G53340	Control gene	At1g61180	R-gene
AT1G33060	Stress response gene	AT1G53440	Control gene	At1g61190	R-gene
AT1G37130	Stress response gene	AT1G54510	Control gene	AT1G61300	R-gene
AT1G47600	Stress response gene	AT1G54620	Control gene	AT1G61310	R-gene
AT1G50140	Stress response gene	AT1G58050	Control gene	AT1G62630	R-gene
AT1G51790	Stress response gene	AT1G58150	Control gene	AT1G63350	R-gene
AT1G53240	Stress response gene	AT1G58160	Control gene	AT3G07040	R-gene
AT1G55010	Stress response gene	AT1G58450	Control gene	AT3G14460	R-gene
AT1G56650	Stress response gene	AT1G60830	Control gene	AT3G14470	R-gene
AT1G59870	Stress response gene	AT1G61370	Control gene	AT3G15700	R-gene
AT1G61360	Stress response gene	AT1G61750	Control gene	AT3G46530	R-gene
AT1G63522	Stress response gene	AT1G61760	Control gene	AT3G46710	R-gene
AT1G64670	Stress response gene	AT1G61770	Control gene	AT3G46730	R-gene

AT1G66280	Stress response gene	AT1G61850	Control gene	AT3G50950	R-gene
AT1G68090	Stress response gene	AT1G61860	Control gene	AT4G10780	R-gene
AT1G69828	Stress response gene	AT1G61860	Control gene	AT4G14610	R-gene
AT1G71220	Stress response gene	AT1G61870	Control gene	AT4G19050	R-gene
AT1G72850	Stress response gene	AT1G61880	Control gene	AT4G27190	R-gene
AT1G73805	Stress response gene	AT1G61970	Control gene	AT4G27220	R-gene
AT1G75560	Stress response gene	AT1G61980	Control gene	AT4G33300	R-gene
AT1G77120	Stress response gene	AT1G62090	Control gene	AT5G04720	R-gene
AT1G78600	Stress response gene	AT1G65300	Control gene	AT5G05400	R-gene
AT1G80460	Stress response gene	AT1G66550	Control gene	AT5G35450	R-gene
AT2G02470	Stress response gene	AT1G67810	Control gene	AT5G43470	R-gene
AT2G03955	Stress response gene	AT1G67840	Control gene	AT5G43730	R-gene
AT2G06530	Stress response gene	AT2G10090	Control gene	AT5G43740	R-gene
AT2G15080	Stress response gene	AT2G21030	Control gene	AT5G45510	R-gene
AT2G17380	Stress response gene	AT2G21040	Control gene	AT5G47250	R-gene
AT2G18880	Stress response gene	AT3G04050	Control gene	AT5G47260	R-gene
AT2G20465	Stress response gene	AT3G09240	Control gene	AT5G47280	R-gene
AT2G21870	Stress response gene	AT3G18160	Control gene	AT5G48620	R-gene
AT2G23620	Stress response gene	AT3G18180	Control gene	AT5G63020	R-gene
AT2G25620	Stress response gene	AT3G19700	Control gene	AT5G66630	R-gene
AT2G27145	Stress response gene	AT3G26540	Control gene	AT1G17600	R-gene
AT2G29045	Stress response gene	AT3G42530	Control gene	AT1G27170	R-gene
AT2G31180	Stress response gene	AT3G44410	Control gene	AT1G27180	R-gene
AT2G32730	Stress response gene	AT3G44490	Control gene	AT1G31540	R-gene
AT2G34600	Stress response gene	AT3G44640	Control gene	AT1G56510	R-gene
AT2G36530	Stress response gene	AT3G44680	Control gene	AT1G56520	R-gene
AT2G38560	Stress response gene	AT3G48790	Control gene	AT1G56540	R-gene
AT2G40030	Stress response gene	AT3G48810	Control gene	AT1G63730	R-gene
AT2G41370	Stress response gene	AT3G53060	Control gene	AT1G63740	R-gene
AT2G43090	Stress response gene	AT3G53590	Control gene	AT1G63750	R-gene
AT2G44920	Stress response gene	AT3G53600	Control gene	AT1G63860	R-gene

AT2G46450	Stress response gene	AT4G08970	Control gene	AT1G63870	R-gene
AT2G48150	Stress response gene	AT4G09360	Control gene	AT1G63880	R-gene
AT3G02400	Stress response gene	AT4G10200	Control gene	AT1G64070	R-gene
AT3G03710	Stress response gene	AT4G10220	Control gene	AT1G65850	R-gene
AT3G04945	Stress response gene	AT4G10460	Control gene	AT1G69550	R-gene
AT3G06050	Stress response gene	AT4G11400	Control gene	AT1G72840	R-gene
AT3G08580	Stress response gene	AT4G11470	Control gene	AT1G72860	R-gene
AT3G09940	Stress response gene	AT4G16080	Control gene	AT2G14080	R-gene
AT3G11410	Stress response gene	AT4G16530	Control gene	AT2G17050	R-gene
AT3G12810	Stress response gene	AT4G16540	Control gene	AT2G17060	R-gene
AT3G14210	Stress response gene	AT4G16550	Control gene	AT3G04220	R-gene
AT3G15950	Stress response gene	AT4G16560	Control gene	AT3G25510	R-gene
AT3G17155	Stress response gene	AT4G17860	Control gene	AT3G44400	R-gene
AT3G18910	Stress response gene	AT4G17970	Control gene	AT3G44480	R-gene
AT3G20550	Stress response gene	AT4G17980	Control gene	AT3G44630	R-gene
AT3G22780	Stress response gene	AT4G18000	Control gene	AT3G44670	R-gene
AT3G23870	Stress response gene	AT4G18020	Control gene	AT3G51560	R-gene
AT3G25265	Stress response gene	AT4G18030	Control gene	AT3G51570	R-gene
AT3G27150	Stress response gene	AT4G18040	Control gene	AT4G08450	R-gene
AT3G28910	Stress response gene	AT4G18360	Control gene	AT4G09360	R-gene
AT3G44380	Stress response gene	AT4G18600	Control gene	AT4G09430	R-gene
AT3G46630	Stress response gene	AT4G26890	Control gene	AT4G11170	R-gene
AT3G48190	Stress response gene	AT4G26920	Control gene	AT4G12010	R-gene
AT3G49810	Stress response gene	AT4G30090	Control gene	AT4G12020	R-gene
AT3G51450	Stress response gene	AT4G32220	Control gene	AT4G14370	R-gene
AT3G52930	Stress response gene	AT4G32230	Control gene	AT4G16860	R-gene
AT3G55320	Stress response gene	AT4G38070	Control gene	AT4G16890	R-gene
AT3G57130	Stress response gene	AT4G38300	Control gene	AT4G16900	R-gene
AT3G59660	Stress response gene	AT5G03720	Control gene	AT4G16920	R-gene
AT3G61190	Stress response gene	AT5G04400	Control gene	AT4G16940	R-gene
AT3G66652	Stress response gene	AT5G13290	Control gene	AT4G16950	R-gene

AT4G02070	Stress response gene	AT5G14300	Control gene	AT4G16960	R-gene
AT4G03560	Stress response gene	AT5G14400	Control gene	AT4G19500	R-gene
AT4G08170	Stress response gene	AT5G14490	Control gene	AT4G19510	R-gene
AT4G09984	Stress response gene	AT5G14560	Control gene	AT4G19520	R-gene
AT4G11290	Stress response gene	AT5G14980	Control gene	AT4G19530	R-gene
AT4G12880	Stress response gene	AT5G14990	Control gene	AT4G36140	R-gene
AT4G14630	Stress response gene	AT5G15000	Control gene	AT4G36150	R-gene
AT4G16845	Stress response gene	AT5G18690	Control gene	AT5G11250	R-gene
AT4G17750	Stress response gene	AT5G31927	Control gene	AT5G17680	R-gene
AT4G19530	Stress response gene	AT5G32450	Control gene	AT5G17880	R-gene
AT4G21600	Stress response gene	AT5G36270	Control gene	AT5G17890	R-gene
AT4G23150	Stress response gene	AT5G36280	Control gene	AT5G17970	R-gene
AT4G24220	Stress response gene	AT5G36780	Control gene	AT5G18350	R-gene
AT4G25200	Stress response gene	AT5G40470	Control gene	AT5G18360	R-gene
AT4G26780	Stress response gene	AT5G40730	Control gene	AT5G18370	R-gene
AT4G29033	Stress response gene	AT5G40740	Control gene	AT5G22690	R-gene
AT4G30340	Stress response gene	AT5G42260	Control gene	AT5G36930	R-gene
AT4G31910	Stress response gene	AT5G42510	Control gene	AT5G38340	R-gene
AT4G33420	Stress response gene	AT5G42840	Control gene	AT5G38350	R-gene
AT4G34710	Stress response gene	AT5G43200	Control gene	AT5G38850	R-gene
AT4G36430	Stress response gene	AT5G43260	Control gene	AT5G40060	R-gene
AT4G38130	Stress response gene	AT5G43360	Control gene	AT5G40100	R-gene
AT4G39850	Stress response gene	AT5G43370	Control gene	AT5G40910	R-gene
AT5G02840	Stress response gene	AT5G43480	Control gene	AT5G40920	R-gene
AT5G04930	Stress response gene	AT5G43490	Control gene	AT5G41540	R-gene
AT5G06720	Stress response gene	AT5G43490	Control gene	AT5G41550	R-gene
AT5G08120	Stress response gene	AT5G43520	Control gene	AT5G41740	R-gene
AT5G09978	Stress response gene	AT5G43520	Control gene	AT5G41750	R-gene
AT5G11670	Stress response gene	AT5G43530	Control gene	AT5G44510	R-gene
AT5G13680	Stress response gene	AT5G43550	Control gene	AT5G44870	R-gene
AT5G15180	Stress response gene	AT5G44060	Control gene	AT5G45050	R-gene

AT5G16990	Stress response gene	AT5G44140	Control gene	AT5G45060	R-gene
AT5G18610	Stress response gene	AT5G44250	Control gene	AT5G45200	R-gene
AT5G20230	Stress response gene	AT5G44480	Control gene	AT5G45210	R-gene
AT5G22500	Stress response gene	AT5G44700	Control gene	AT5G45230	R-gene
AT5G24530	Stress response gene	AT5G44760	Control gene	AT5G45240	R-gene
AT5G26860	Stress response gene	AT5G44770	Control gene	AT5G45250	R-gene
AT5G34850	Stress response gene	AT5G44910	Control gene	AT5G45260	R-gene
AT5G37510	Stress response gene	AT5G44930	Control gene	AT5G46260	R-gene
AT5G39740	Stress response gene	AT5G45490	Control gene	AT5G46270	R-gene
AT5G41360	Stress response gene	AT5G45530	Control gene	AT5G46450	R-gene
AT5G42980	Stress response gene	AT5G45570	Control gene	AT5G46470	R-gene
AT5G44080	Stress response gene	AT5G45610	Control gene	AT5G46490	R-gene
AT5G45220	Stress response gene	AT5G45740	Control gene	AT5G46510	R-gene
AT5G46420	Stress response gene	AT5G45750	Control gene	AT5G46520	R-gene
AT5G47390	Stress response gene	AT5G46830	Control gene	AT5G48770	R-gene
AT5G48905	Stress response gene	AT5G47170	Control gene	AT5G49140	R-gene
AT5G50720	Stress response gene	AT5G47510	Control gene	AT5G51630	R-gene
AT5G52605	Stress response gene	AT5G50620	Control gene	AT5G58120	R-gene
AT5G54230	Stress response gene	AT5G62990	Control gene	AT1G47370	R-gene
AT5G55920	Stress response gene	AT5G63070	Control gene		
AT5G57970	Stress response gene	AT5G67630	Control gene		
AT5G59520	Stress response gene	AT1G59830	Control gene		
AT5G61560	Stress response gene				
AT5G63320	Stress response gene				
AT5G64520	Stress response gene				
AT5G66130	Stress response gene				

Table S3. Treatments considered in the metastudy and the studies from which they were derived. For the perturbation treatments, gene expression under the perturbation was compared to that of independent, unperturbed plants.

Condition	Array Express Repository ID	# *R* Genes	# Control Genes	# Stress Response Genes	Sampled Tissue	Experimental Setup (when not stated, plants are on soil)	Reference
B. graminis	E-GEOD-12856	16	26	35	Rosettes	At growth stage 3.90, harvested 12 hpi	Jensen *et al*., 2008
E. cichoracearum	E-GEOD-431	48	24	61	leaves	Infected at 21 d, harvested 3 dpi	Nishimura *et al*., 2003
Hyaloperonospora parasitica	E-GEOD-18329	60	54	95	rosettes	Infected at 7 d, harvested 4 dpi	Bhattari *et al*., 2010
Pseudomonas syringae* pv. DC3000 with *avrRpm1	E-GEOD-6176	14	10	32	leaf	At growth stage 3.90, harvested 4 hpi	None associated
Pseudomonas syringae* with *AvrRpt2	E-GEOD-58954	61	57	89	Mature leaf	Harvested 6 h post infection	None associated
Pseudomonas syringae* with *AvrRpt2	E-GEOD-6556	41	33	90	leaf	Infected at 35 d, harvested 2 dpi	Zhang *et al*., 2007
Pseudomonas syringae* pv. tomato with *HopZ1a	E-GEOD-21920	52	33	87	Rosette leaf	No information	None associated
***Pseudomonas syringae* pv. tomato**	E-GEOD-21920	26	12	53	Rosette leaf	No information	None associated
**flg22**	E-NASC-76	8	18	24	seedlings on agar	At 10 d on agar; flg22 treatment for 1 and 3 h	Denoux *et al*., 2008
**SA**	E-TABM-51	44	22	70	Rosette leaves	At 42-49 d, 4, 28, or 52 h SA	Van Leeuwen *et al*., 2007
**SA**	E-GEOD-22942	25	17	87	Rosette leaves	At 4d, 40 min SA; harvested 10 h post treatment	None associated
**4°C**	E-MEXP-1345	42	36	78	leaf discs	At 45 d, 22-26 h 4°C exposure	Bieniawska *et al*., 2008
**4°C**	E-TABM-52	40	30	79	bolting rosette	At bolting, to 4°C for 14 d	Hannah *et al*., 2006
**37°C**	E-GEOD-11758	52	39	89	Rosette leaves	At 35 d, 1 h heat shock	Sugio *et al*., 2009
**Drought**	E-GEOD-40061	52	38	106	Rosette leaves	At 21 d, 10 d drought stress	Pandey *et al*., 2013
**Light**	E-MTAB-392	72	53	140	leaf	At 24 d, 30 – 120 min excess light (1300 µmol photons/m^{2}s)	None associated
**Ozone**	E-MEXP-342	17	11	35	Whole plant	At 10d, 500ppb ozone	Van Aken *et al*., 2009

Figure S1. Clinal variation in basal expression with latitude or population in the Swedish RNA-seq dataset. The proportion of *R*-genes and control genes that are expressed significantly higher in the northern than the southern Swedish population, non-differentially expressed between populations, or expressed significantly higher in the southern than the northern Swedish population.

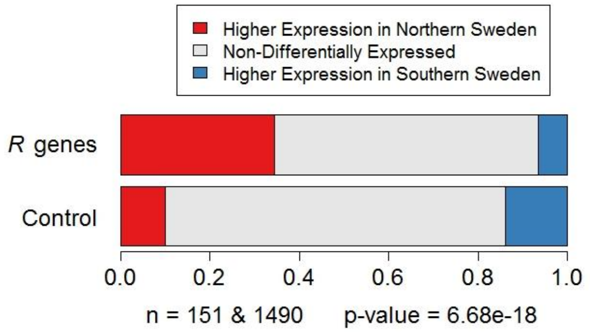

Notes Table S3 Supplementary References for Table S2.

Bhattarai K, Atamian H, Kaloshian I & Eulgem T. 2010. WRKY72-type transcription factors contribute to basal immunity in tomato and *Arabidopsis* as well as gene-for-gene resistance mediated by the tomato R gene Mi-1. *Plant J. Cell Mol. Biology* **63**: 229–40.

Bieniawska Z, Espinoza C, Schlereth A, Sulpice R, Hincha D, Hannah M. 2008. Disruption of the *Arabidopsis* circadian clock is responsible for extensive variation in the cold-responsive transcriptome. *Plant physiology* **147**: 263–79.

Denoux C, Galletti R, Mammarella N, Gopalan S, Werck D, Lorenzo GD, Ferrari S, Ausubel FM, Dewdney J. 2008. Activation of Defense Response Pathways by OGs and Flg22 Elicitors in *Arabidopsis* Seedlings. *Molecular Plant* **1**.

Hannah M, Wiese D, Freund S, Fiehn O, Heyer A, Hincha D. 2006. Natural genetic variation of freezing tolerance in Arabidopsis. *Plant Physiology* **142**: 98–112.

Jensen M, Hagedorn P, Torres-Zabala M, Grant M, Rung J, Collinge D, Lyngkjaer M. 2008. Transcriptional regulation by an NAC (NAM-ATAF1,2-CUC2) transcription factor attenuates ABA signalling for efficient basal defence towards *Blumeria graminis* f. sp. hordei in *Arabidopsis*. *Plant J. Cell Mol. Biology* **56**: 867–80.

Nishimura MT, Stein M, Hou B-HH, Vogel JP, Edwards H, Somerville SC 2003. Loss of a callose synthase results in salicylic acid-dependent disease resistance. *Science* **301**: 969–72.

Pandey N, Ranjan A, Pant P, Tripathi R, Ateek F, Pandey H, Patre U, Sawant S. 2013. CAMTA 1 regulates drought responses in Arabidopsis thaliana. *Bmc Genom*. **14**: 216.

Sugio A, Dreos R, Aparicio F & Maule A. 2009. The Cytosolic Protein Response as a Subcomponent of the Wider Heat Shock Response in Arabidopsis. *Plant Cell Online* **21**: 642–654.

Van Aken O, Zhang B, Carrie C, Uggalla V, Paynter E, Giraud E, Whelan J. 2009. Defining the Mitochondrial Stress Response in Arabidopsis thaliana. *Mol. Plant* **2**: 1310–1324.

Van Leeuwen H, Kliebenstein D, West M, Kim K, Poecke R, Katagiri F, Michelmore R, Doerge R, Clair D. 2007. Natural variation among *Arabidopsis thaliana* accessions for transcriptome response to exogenous salicylic acid. *Plant Cell* **19**: 2099–110.

Zhang Z, Li Q, Li Z, Staswick P, Wang M, Zhu Y, He Z. 2007. Dual regulation role of GH3.5 in salicylic acid and auxin signaling during *Arabidopsis-Pseudomonas* syringae interaction. *Plant Physiology* **145**: 450–64.
